# Comparison of the shaping characteristics of Neolix and Protaper Universal systems in preparation of severely-curved simulated canals

**DOI:** 10.4317/jced.53476

**Published:** 2017-04-01

**Authors:** Maryam Forghani, Maryam Hezarjaribi, Hamidreza Teimouri

**Affiliations:** 1 Dental Materials Research Center, Mashhad University of Medical Sciences, Mashhad, Iran; 2 Postgraduate student of Operative Dentistry, Dental Research Center, Mashhad University of Medical Sciences, Mashhad, Iran; 3 Private Practice, Tehran, Iran

## Abstract

**Background:**

The aim of this *in vitro* study was to compare the shaping characteristics of two types of nickel-titanium endodontic file systems with regard to the following parameters: Canal straightening, Canal deviation and Instrumentation time.

**Material and Methods:**

Fifty severely curved canals simulated in resin blocks were prepared to an apical size 25 using Protaper Universal orNeolix systems (n=25 canals/group). The angle of canal curvature was determined before and after instrumentation. Pre- and post-operative images were superimposed to determine any canal deviation. The instrumentation time was also recorded. The data were statistically analyzed by using independent sample t-test and the Mann-Whitney U-test.

**Results:**

There were no significant differences between the groups in terms of the change in canal angle (*P* > 0.05). The Neolix system produced less canal deviation at 7 of the 12 measuring points (*p*<0.05). The Neolix system also required less instrumentation time (*p*<0.05).

**Conclusions:**

Both rotary systems were capable of maintaining the original curvature of the root canal; however, the Neolix system resulted in less canal deviation as well as shorter instrumentation time.

** Key words:**Apical foramen,canal transportation, nickel-titanium instruments, Protaper Universal, root canal preparation.

## Introduction

Root canal preparation is expected to preserve the original canal curvature - a conical shape tapering from crown to apex (1). However, maintaining the original canal direction and avoiding canal aberrations such as ledges, zips, and perforations remains challenging, especially when preparing curved canals (2). Nickel-titanium (NiTi) instruments were introduced to endodontics toshape root canals more efficiently and reduce the incidence of procedural errors; since they have 2-3 times greater flexibility-compared tostainless steel instruments (3). Besides enhancing the overall shaping quality, NiTirotary systems reduce operator fatigue and treatment time (4).

NiTi rotary systems continue to evolve in terms of design, promising optimized cutting and shaping efficiency. During the recent years, a number of advancements in thermo-mechanical treatment and manufacturing technologies have led to optimization of the microstructure ofNiTi alloys (5). Neolix (Neolix, châtres-la-Forêt, France) is a newly introduced NiTi rotary system with full rotary motion that consist of one C1 file for coronal enlargement and three A1 files (with tip size range of #20, #25 and # 40) allowing for canal shaping down to the apex. Neolix files are generated using a newly developed wire-cut electrical discharge machining (WEDM) process. The manufacturer claims that this process produces a rough surface, with abrasive properties resulting in faster root canal preparation. Furthermore, the manufacturer asserts that the appropriate heat treatment delivered by these files results in higher flexibility (6,7).

The present study was performed to evaluate the ability of the Neolix system in maintaining the original profile of the canal and also in terms of canal preparation time when dealing with severely-curved simulated canals. Protaper Universal (DentsplyMaillefer, Ballaigues, Switzerland) system was used as a benchmark system for comparison; as it often serves as a golden standard to which new file systems are compared (8-11). The null hypothesis was that there would be no significant differences in terms of canal straightening, centering ability and preparation time between the tested NiTi rotary file systems.

## Material and Methods

The research protocol was approved by the Vice Chancellor for Research of Mashhad University of Medical Sciences (No.941068). 50 transparent resin blocks with simulated, standardized curved root canals (Acadental, Kansas, USA) with curvature of 45°, a taper degree of 2°, having apical diameter of 0.15 mm and length of 17 mm,were randomly assigned to two groups according to the experimental instrumentation system (n=25): the Neolix group and the Protaper Universal group. The blocks were marked at three reference points to facilitate the super imposition of the pre-and post-operative images.

Prior to any instrumentation procedure, the blocks were placed on a customized stand, then fixed under a stereomicroscope (AM413T Dino-Lite Pro, AnMo Electronics Corporation, New Taipei, Taiwan), and the preoperative images were obtained. A glide path was established in each canal using a stainless steel #15 K-file (Dentsply, Maillefer) to the working length (WL), which was set at the terminus of the simulated canal. Instrumentation was performed by a single experienced operator using an endodontic electric motor (Silver Reciproc, VDW GmbH, Germany) in accordance with the manufacturer’s recommendations for speed and torque. As for Protaper Universal system, the SX (1/2 of the WL), S1 (17/02), S2 (20/04), F1 (20/07) and F2 (25/08) files were used. The Neolix-group files comprised C1 (1/2of the WL), A1 (20/08) and A1 (25/08).

After performing three in-and-out movements, the flutes of the instruments were cleaned and canal irrigation was conducted with 2 ml of normal saline (Sodium Chloride 0.9%) using a 27-gauge needle. The files were removed once they reached the working length and rotated freely.

Each instrument was used to prepare five canals and after each passage was evaluated under X2.5 magnification to check for any deformation. The length of time spent on canal preparation included active instrumentation, sequential change of instruments, cleaning of the flutes and irrigation, which was recorded with a chronometer. During all preparation procedures, RC LUBE (Master-Dent, USA) was used as lubricant. After the final irrigation, the post-operative images were obtained in the manner described earlier.

Pre- and post-operative images were superimposed digitally (Adobe Photoshop version cs6), precisely overlapping the reference points of each pair of images.

Deviation of the root canals was determined according to the study by Hiranus *et al.* (11). The distance between the canal wall on pre-and post-operation images was measured at 1 mm intervals on both the mesial and distal sides of the canal.

The first measuring point was at the apical foramen and the last was marked at 12 mm from the apical foramen (a total of 12 measuring points along the canal). Measurements were made using Microstructural Image Processingsoftware (MIP4, Nahamin-Pardazan Asia Co., Iran) by an experienced clinician blinded in respect to all experimental groups. Canal deviation was calculated as the difference between the outer side transportation and the inner side transportation. The absolute values of the calculation as well as the direction of the deviation (outer or inner) were also recorded.

The angle of curvature was determined for each canal before and after instrumentation in accordance with the Schneider method (12).

-Statistical analysis:

Statistical evaluations were performed using the SPSS software. The Shapiro-Wilk test was employed to verify the normality of the data. Changes in the angle of canal curvature were compared between the groups using the Mann-Whitney U-test. Canal deviation values were compared using the independent sample t-test and Mann-Whitney U-test. The instrumentation time was also compared using the independent sample t-test. A significance level of 0.05 was set for all statistical analysis.

## Results

**Figure 1:**
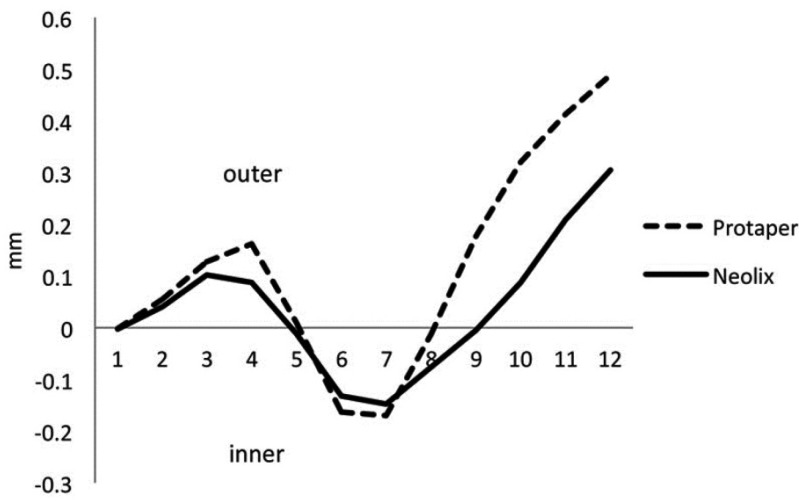
Extent and Direction of canal deviation (mm) at different measurement levels of canals.

Both rotary systems produced deviations at all canal levels evaluated (Fig. 1). The Protaper Universal group showed greater ‘mean canal deviation’ at 7 of the 12 measuring points (*P*<0.05) but the Neolix group revealed the same greater index at only 1 measuring point (*P*=0.001).

With regards to the preparation time, the Neolix system prepared the canals significantly faster (136/92 seconds) than its Protaper counterpart (194/54 seconds) (*P*<0.001) ( Table 1).

**Table 1:**
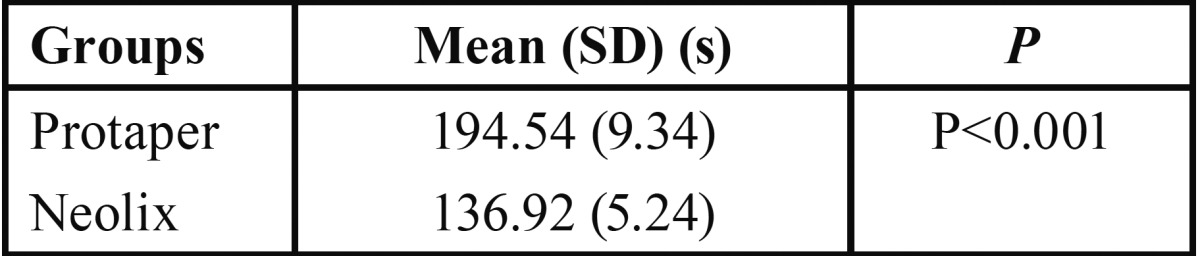
Mean preparation time in seconds (s) across different groups.

The data concerning the mean (SD) of the difference between the angle of canal curvature before and after instrumentation are presented in  Table 2. There was no significant difference between the groups with respect to canal straightening (*P*=0.808).

**Table 2:**
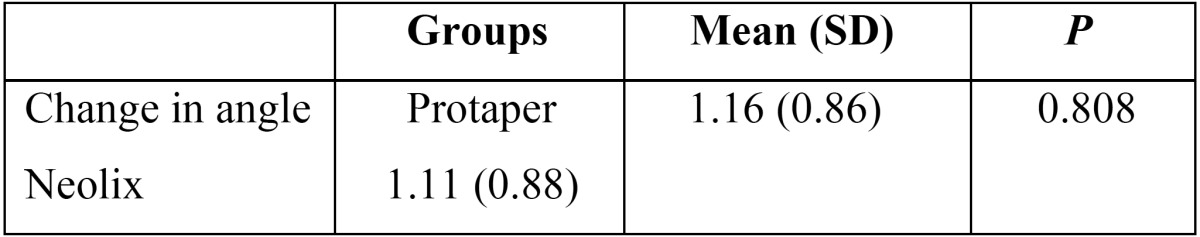
Change in angle of canal curvature (degree) in experimental groups.

## Discussion

The present study investigated the ability of two NiTi endodontic file systems in shaping severely-curved simulated canals based on canal straightening, canal deviation and instrumentation time. With respect to canal deviation and preparation time, the Neolix system performed better compared to the Protaper Universal system.

The current study chose simulated canals in resin blocks because the standardization of canal morphology is a decisive factor when comparing the shaping ability of different instruments. Furthermore, resin blocks can be easily photographed and evaluated before and after canal instrumentation (13). This method, however, has some potential shortcomings such as the possible differences in the mechanical properties of the resin and those of the human dentin. Yet, because the conditions are almost identical for the different rotary systems applied, the results obtained using resin blocks could be validated for clinical situations (14).

In this study, the apical preparation diameter was standardized using instruments with a tip diameter equivalent to size 25. Hoppe *et al.* (15) reported that the enlargement of the canal to size 25 could accommodate good cleaning. Increasing the apical preparation size may increase the risk of canal transportation because it could result in decreased flexibility of the instruments (16,17).

It is desirable to maintain the original shape and position of the apical foramen during the root canal preparation (1) because any changes in these parameters could exert a negative impact on the efficacy of the seal that is provided by filling (18). A previous study revealed that an apical transportation greater than 0.3 mm can lead to loss of apical seal (18).

In this study, no significant differences were observed between the groups in relation to transportation of apical 2mm of the canals. But at 3mm level, the Protaper system produced significantly more transportation compared to the Neolix system. The range of apical transportation was found to be between 0.002-0.128 and 0.003-0.102 in Protaper and Neolix groups respectively. It is fair to assume that the resultant apical transportation in both groups would not compromise the apical seal (17).

The mean difference between the pre- and post-operative angle in the Protaper group was 1.16±0.86, which is in agreement with the findings of previous studies (10,19). The Neolix system led to 1.11±0.88 amount of change in angle. The changes in the canal curvature, which followed canal preparation using Protaper and Neolix rotary systems, were not statistically significant and both systems achieved good results. This finding might be attributed to the noncutting tips that these instruments have (7,20,21). A noncutting tip can work with minimal apical pressure and functions only as a guide for easy penetration (22).

In this study, Neolix files showed greater centering ability compared to Protaper files. One explanation may be the improved flexibility of the Neolix instruments. The flexibility of endodontic instruments depends on the metallurgic properties of their alloy (its chemical composition and thermo-mechanical properties) as well as the geometric shape and size of the instruments (23,24). Neolix files do not show the usual metallic memory and tendency to rapidly return to straight position. The manufacturer has claimed that this special feature is due to the use of a newly developed wire-cut electrical discharge machining (WEDM) process and an appropriate heat treatment in manufacturing of these files (6,7).

As far as the Protaper Universal system is concerned, the greater number of instruments and the greater amount of time the files are used working inside the root canal may also affect its ability in deviation the original curvature of the root canals in comparison with the Neolix system.

The preparation time is dependent on different factors such as the applied technique, the number of instruments, and the operator’s experience (13). In the present study, the Neolix system was significantly faster than the Protaper files, which was probably related to the number of files used in each system. Furthermore, the manufacturer of Neolix files claims that these instruments with rough surface and abrasive properties are faster in root canal preparation. Reduced instrumentation time, especially in case of posterior teeth with complex root canal anatomy can decrease chair time and improve the overall health care.

## Conclusions

Within the limitations of the present study, both Protaper Universal and Neolix instruments proved to be relatively safe in preparation of severely curved canals. However, Neolixinstruments produced less canal deviationand required shorter preparation time compared to Protaper Universal system.
